# Fifty-Year Trend Towards Suppression of *Wolbachia-*Induced Male-Killing by Its Butterfly Host, *Hypolimnas bolina*

**DOI:** 10.1673/031.011.9201

**Published:** 2011-07-23

**Authors:** Wataru Mitsuhashi, Hiroshi Ikeda, Masahiko Muraji

**Affiliations:** ^1^ National Institute of Agrobiological Sciences, Tsukuba, Ibaraki 305–8634, Japan; ^2^ Itami Senior High School, Gyouki-cho 4–1, Itami, Hyougo 664–0857, Japan

**Keywords:** evolutionary biology, intracellular symbiont, reproductive alteration, sex ratio

## Abstract

Some intracellular symbionts of arthropods induce a variety of reproductive alterations in their hosts, and the alterations tend to spread easily within the host populations. A few cases involving the spread of alteration-inducing *Wolbachia* bacteria in natural populations with time have been reported, but the investigations on the increasing trend in counteracting the bacterial effect on hosts in natural populations (i.e., increased resistance in hosts against the alterations) have been limited. In the present study, the prevalence of an alteration, killing of male *Hypolimnas bolina* (L.) (Lepidoptera: Nymphalidae) butterflies by their inherited *Wolbachia* strain in the wild in Japan, was surveyed over a continuous 50-year period, which is far longer than ever before analyzed in studies of dynamics between reproductive alteration-inducing symbionts and their host arthropods. Thus, the results in this study provide the first instance of a long-term trend involving a change in reproductive alteration; and it strongly suggests a change in the opposite direction (i.e., suppression of male-killing) in natural populations. This change in the current combination of the *Wolbachia* and butterflies appears to be dependent upon the host taxon (race).

## Introduction

Some intracellular symbionts are known to alter the reproduction of their host arthropods, but information on the evolutionary biology of the relationship between hosts and their reproductive parasites in natural populations is limited. A few instances of the spread of *Wolbachia* bacteria in natural populations have been reported (Turelli and Hoffmann 1991; Hoshizaki and Shimada 1995; Hiroki et al. 2005), but studies demonstrating the reactive dynamics of hosts in natural populations (i.e., increased suppression of the alterations by host) are limited. Responses by hosts to mitigate the alterations on symbionts in natural populations have been examined several times, and the evolutionary stability of male-killing in several insect species has been reported (Hurst et al. 2001; Jaenike and Dyer 2008).

Some lines of the great egg-fly, *Hypolimnas bolina* (L.) (Lepidoptera: Nymphalidae), show a female-biased sex ratio and harbor the *Wolbachia* strain *w*Boll (Dyson et al. 2002; Mitsuhashi et al. 2004), but some lines of this species with a normal sex ratio also harbor *w*Boll (Mitsuhashi et al. 2004; Charlat et al. 2005). However, the increase in hatch rates and the recovery of the sex ratio towards normal after antibiotic treatment of this species indicate that the *w*Boll strain causes male-killing (Mitsuhashi et al. 2004; Charlat et al. 2007a). Findings indicate that the normal sex ratio in lines harboring *w*Boll is maintained by the suppression of male-killing by a factor in the host insect (Hornett et al. 2006). This was based on the results of laboratory-scale experiments of several crosses between lines expressing male-killing trait and lines with both a normal sex ratio and *w*Boll. In addition, an extremely rapid change in sex bias was reported for a Polynesian population of *H. bolina,* with a switch from a male:female sex ratio of 1:100 to 1:1 within 1 year, which implies a very rapid spread of the suppression of male-killing in the population (Charlat et al. 2007b). However, monitoring this change was very limited (2 years), and further observations over a longer time period are necessary to determine whether the new sex ratio trend will continue.

When analyzing the relationship between reproductive parasites and their host arthropods in the wild, continuous data obtained from long-term studies are important in many study cases; however, to date, only analyses over a short time period (at longest 10 years) or comparisons of data between two periods separated by a long unexamined period have been performed (Hoshizaki and Shimada 1995; Hiroki et al. 2005; Hornett et al. 2009).

To investigate whether a change in the prevalence of male-killing has already occurred in natural populations, Japanese adult butterflies of *H. bolina* over a long period (the past 50 years) were examined. We first investigated the prevalence of the suppression of male-killing in this species in recent years (2003–2009) in Japan. Second, data including the date and locality in which the male(s) and/or female(s) of adult *H. bolina* were caught or witnessed over the past approximately 50 years in Japan were analyzed to investigate the status of the sex ratio during this period.

Lines with *w*Boll and a normal sex ratio (hereafter referred to as ‘male-killing suppression lines’) in *H. bolina* have recently been found in several areas of Asia (Mitsuhashi et al. 2004; Charlat et al. 2005; Hornett et al. 2006); one study found that male-killing suppression was prevalent in the Kota Kinabalu population in Malaysia (Charlat et al. 2005), but in the other two reports the presence of male-killing suppression was shown only by examining small butterfly samples in a few small areas. Therefore the prevalence of male-killing suppression lines and lines showing female bias due to male-killing (hereafter referred to as male-killing susceptible lines) in Asian populations in recent years remains unclear.

**Table 1.**
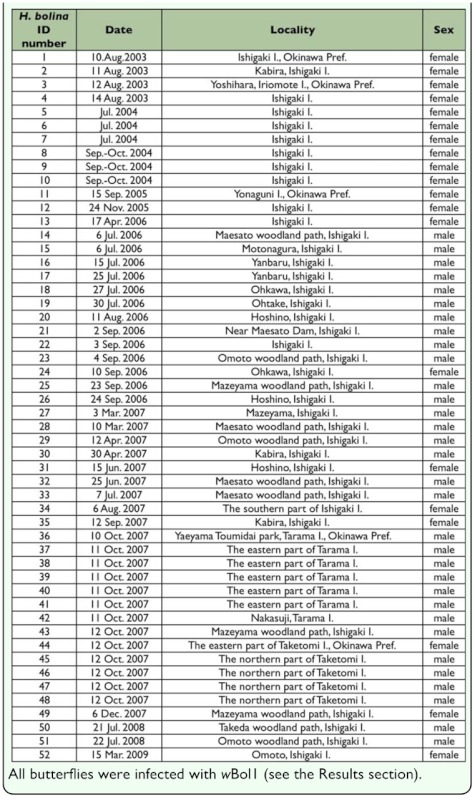
Data of captures of *Hypolimnas bolina* adults in the field, which were used for diagnostic PCR for *Wolbachia* infection.

*H. bolina* shows clear geographical variations in the color and spot patterns on its wings, and is thus divided into several races; among them the race, bolina, and race group, rarik, appear similar. However, mitochondrial DNA sequences show that all the races are genetically closely related (Charlat et al. 2005). Every race has been reported to express a female-biased sex ratio (Fukuda and Nicho 1988; Mitsuhashi et al. 2004). Japan is a unique area with regard to *H. bolina* because all races of the species appear, although races kezia and philippensis are predominant, and race group rarik has been found infrequently (Fukuda et al. 2005; Shirouzu 2006). Most *H. bolina* are migrants from tropical or subtropical areas of neighboring Asian countries, such as the Philippines, southern China, and the Indochinese Peninsula (Shirouzu 1960; Kawazoe and Wakabayashi 1976; Fukuda et al. 1983; Fukuda and Nicho 1988), although their offspring frequently occur in Japan, and sometimes different races interbreed in Japan and thus offspring with hybrid wing patterns are collected there. *H. bolina* is very rare in Japan during the winter. It has a tendency to migrate long distances (Kawazoe and Wakabayashi 1976; Fukuda and Nicho 1988; Corbet and Pendlebury 1992), so movement among the southern areas of the Japanese archipelago is thought to occur easily. In addition, seasonal southwesterly winds and the strong winds of frequent typhoons are considered important in bringing large numbers of *H. bolina* to southern Japan every year (Fukuda and Nicho 1988). Therefore, analyses of the respective races of *H. bolina* in Japan are representative of analyses of these races in their primary distributional areas (e.g. the Philippines for philippensis and southern China for kezia).

This study provides for the first time the results of an analysis over a long, continuous period of natural populations of a host arthropod that have reacted effectively against reproduction alteration by symbionts. Furthermore, the existence of a third *Wolbachia* strain in *H. bolina* is reported.

## Materials and Methods

### Insects

**Figure 1.**
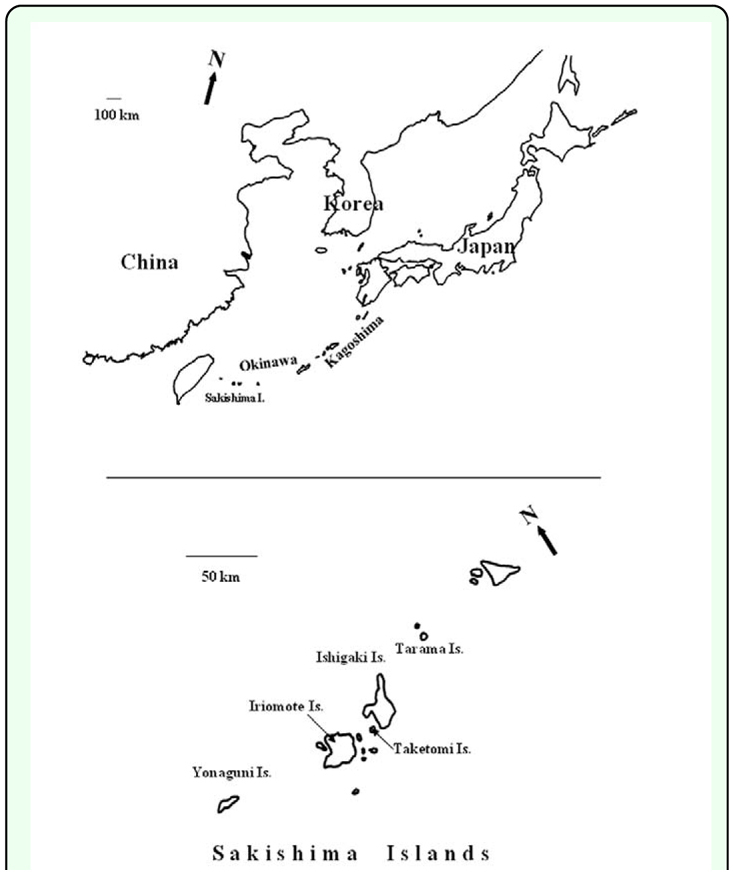
Map of the area (Japan) covered in the present study. The Sakishima Islands, Okinawa Pref., one of the major study areas, are shown in detail in the lower panel. High quality figures are available online.

The *H. bolina* adults used in the present study were caught in fields of 5 islands in Okinawa Prefecture, Japan (Figure 1, Table 1). During the initial period of the present study, females were selectively caught (*H. bolina* of ID numbers 1 –12).

### Rearing for phenotype determination of lines

The prevalence of male-killing suppression lines in *H bolina* in Japan over the recent period (2003–2009) was surveyed by examining the sex-ratio phenotype of lines, by conducting diagnostic PCR for *Wolbachia* infection, and by comparing the *Wolbachia* gene sequences in our samples with the sequences of *Wolbachia* strain *w*Boll, as described below.

Nine captured female adults *(H bolina* ID numbers 5, 8, 11, 31, 34, 35, 44, 49, and 52; see Table 1) were reared on 3–4 % (w/v) sucrose solution or ion-supplemented water (Pocari Sweat; Otsuka Pharmaceutical) containing no antibacterial ingredients. They laid eggs on their food plants (e.g. *Ipomoea batatas, Achyranthes bidentata,* and *Althaea rosea)* without mating after capture. In most cases rearing was conducted at 25° C, although in some cases it was conducted at room temperature. The numbers of male and female F1 adults that emerged were counted to determine whether each *H. bolina* line expressed a male-killing trait.

### DNA extraction

DNA was extracted from all of the wild *H. bolina* adults shown in Table 1 (52 individuals; 32 males and 20 females) and some F1 adults of some of the wild female adults (5 F1 females of *H. bolina* ID number 8; 2 F1 females of ID number 11; and 2 F1 males and 1 F1 female of each of ID numbers 31, 34, 35, 44, 49, and 52), as described below, for diagnostic PCR for *Wolbachia* and for the DNA sequencing of some genes of *Wolbachia*. The whole abdomen was removed from each live adult *H. bolina* and immediately immersed in 100% acetone in a vial (1 sample per vial) and preserved at room temperature until tissues were excised from the abdomen as described below (exception: the abdomen of 1 *H*. *bolina* was immersed in 99.5% ethyl alcohol and preserved at 4° C). In some cases, tissues were excised immediately from the abdomens without such preservation.

The ovaries were excised from each female abdomen in autoclaved phosphate-buffered saline (0.01 M Na_2_HPO_4_-NaH_2_PO_4_, 0.15 M NaCl) with forceps under a binocular microscope on a clean-bench (Type PCV; HITACHI Ltd., www.hitachi-hta.com). From each male abdomen, several milligrams of tissues consisting of fat bodies (some attached by tracheae) and Malpighian tubes were excised on the clean-bench. Each excised tissue sample was preserved in a 1.5-ml microcentrifuge tube at -30° C or -20° C until its next use. Each of the tissue samples from abdomens collected before 2006 was homogenized using a sterile micropestle in 150 µl of extraction buffer (0.1 M Tris-Cl, 10 mM EDTA, 0.5% SDS) and incubated with 54.5 µg proteinase K for 5 h at 37° C; phenol/chloroform extraction of DNA was then performed. For samples collected after 2005, on the other hand, DNA was extracted by using a DNA purification kit (DNeasy (Blood and) Tissue Kit; QIAGEN Sciences, www.qiagen.com) in accordance with the manufacturer's instructions.

### Diagnostic PCR for *Wolbachia* infection

A diagnostic PCR assay was performed on each DNA sample from the 52 adults shown in Table 1 and their 25 F1 adults mentioned above to detect *Wolbachia* by using the *Wolbachia* 16S rRNA gene-specific primers (O'Neill et al. 1992), the thermal cycle conditions of O'Neill et al. (1992), and a PCR kit (rTaq DNA Polymerase Kit; Toyobo, www.toyobobiologics.com). The positive control sample used in each PCR assay was a DNA sample of *Wolbachia* (*w*Boll) from *H. bolina* that had been prepared in a previous study (Mitsuhashi et al. 2004) or that of *Wolbachia* from *Hishimonoides sellatiformis* (Hemiptera) (Mitsuhashi et al. 2002). Sterile distilled water was used as a negative control sample.

### Sequencing

First, 5 genes (*gatB, coxA, hcpA, ftsZ,* and *fbpA*) for multilocus sequence typing (MLST) of *Wolbachia* (Baldo et al. 2006) in each of 12 samples (*H. bolina* ID numbers 5, 8, 11, 28, 30, 31, 33, 34, 35, 44, 49, and 52 in Table 1) were sequenced as described below; those of one individual (*H. bolina* ID number 44) were sequenced by PCR clone sequencing and those of the other 11 samples were sequenced directly. The primer sequences for the amplification of these genes for sequencing were the same as those used in the MLST protocols (Baldo et al. 2006), and the PCR thermal cycle conditions were according to the standard method (http://pubmlst.Org/wolbachia/info/protocols.shtml), except that a period of 2 min 30 s at 94° C instead of 2 min was adopted in the first step. A high-fidelity PCR kit (Pyrobest DNA Polymerase Kit; Takara, www.takarabio.com) was used for the clone sequencing, and either the rTaq DNA Polymerase Kit (Toyobo) or TAKARA LA PCR Kit ver. 2.1 (Takara) was used for direct sequencing. Negative control PCRs were performed in all the PCRs for these genes.

*GatB* and *ftsZ* in MLST (Baldo et al. 2006) of each of the 40 other wild individuals (Table 1) were also directly sequenced, as described below. The sequences of these 2 genes (together as a set) differentiate strain *w*Boll from other known *Wolbachia* strains in the GenBank and MLST databases; therefore, analysis of the sequences of these 2 genes would help to determine if the *Wolbachia* sample analyzed was *w*Boll or another strain. The primer sequences and thermal cycle conditions of the PCR were the same as those above. From these direct sequences, clear “double peak(s)” in the *gatB* and *ftsZ* chromatograms from only one *H bolina* sample (*H bolina* ID number 2) were found, indicating the presence of heterogeneous sequences, and thus the presence of a coexisting *Wolbachia* strain (or strains). Therefore, PCR and subsequent clone sequencing (by the protocols described above and below) of the 5 genes of *H. bolina* ID number 2 were carried out to isolate the different sequences of the genes, and PCRs of 3 other genes (*coxA, hcpA,* and fbpA) of H. bolina ID number 2 and subsequent direct sequencing were also carried out according to the protocols described above.

In addition, *wsp* (Zhou et al. 1998) was amplified from each of 3 DNA samples from *H. bolina* ID numbers 5, 8, and 11 and sequenced by PCR clone sequencing, and in the sample from *H. bolina* ID number 8, a wider region of *ftsZ* (Werren et al. 1995) than that used in the MLST was also sequenced directly. The conditions of the PCRs for the *wsp* and *ftsZ* were in accordance with the method of Mitsuhashi et al. (2002).

The PCR products were purified using a MinElute Gel Extraction Kit (QIAGEN), Alkaline Phosphatase Exonuclease I (shrimp) (Takara), a Suprec-02 spin-filter (Takara), or a Takara DNA Fragment Purification Kit (Takara). For cloning, some PCR products were cloned into pUC118 (Takara) or pUC18 (Takara) vectors and then used to transform *Escherichia coli* JM 109 or DH5α competent cells. The plasmids in the cells were purified using a Miniprep DNA Purification Kit (Takara), or a TaKaRa MiniBEST Plasmid Purification Kit ver.2.0 (Takara). Then 5 or 6 clones of the PCR products (5 clones; each of the 6 genes of *H. bolina* ID number 8: 6 clones; each of the 5 genes of *H*. *bolina* ID number 2 and the *wsp* of *H*. *bolina* ID numbers 5 and 11) were sequenced. An ABI PRISM BigDye Terminator v3.1 Cycle Sequencing Kit (Applied Biosystems Inc., www.appliedbiosystems.com), Sephadex G50 DNA Grade F (GE Healthcare Bio-Science Corp., www.gehealthcare.com/), and ABI PRISM 3100 Genetic Analyzer (Applied Biosystems) or 3730x1 DNA Analyzer (Applied Biosystems) were used in sequencing. Sequencing primers were as follows: for clone sequencing, M13-47 and RV-M primers or pMD18F and pMD18R primers; for direct sequencing, the same primers as used in the amplification of genes by PCR. The internal primer sequences used for the complete direct sequencing of both strands of *wsp* and *ftsZ* (Werren et al. 1995) were 5′-TGAAGATATGCCTATCACTCC-3′ and 5′-GAGTGATAGGCATATCTTCAA-3′ for *wsp,* and 5′-AGATACACTTATTGTCATTCC-3′ and 5′-TAGAGTCATATCCACCAC-3′ for *ftsZ*.

### Phylogenetic analysis

The phylogenetic relationship between *Wolbachia* strains were analyzed using concatenated sequence data set for the five MLST loci. The sequences were aligned with CLUSTAL W version 1.7 (Thompson et al. 1994). All positions including gaps were then deleted, leaving 1650 bases for use in the analysis. The aligned sequences were analyzed using MEGA version 3.1 software (Kumar et al. 2004) to generate phylogenetic trees based on the neighbor-joining method.

### Analysis of data of wild adult *H. bolina* caught or witnessed over the past 50 years in Japan

To estimate changes in (or stability of) the frequency of male-killing susceptible or suppression lines over time in Japan, the sex ratio of *H. bolina* adults caught or witnessed in the wild in Japan during the past 50 years was analyzed. The data were all those available from Shirouzu (2005) and Chouken Publishing (2005, 2006), as well as unpublished data obtained by us. Shirouzu (2005) listed the validated capture or witness data from 1917 through 2003 and Chouken Publishing (2005, 2006) listed validated data reported in 2004 and 2005. Unpublished data obtained by us were capture or witness data from 2003 through 13 October 2007. Each datum includes the number of each sex of wild adult(s) caught or witnessed, the locality in Japan, and the entire or part of the following information: the date (day, month, year) of capture or witness, taxon (race discrimination), photo(s) of the adult(s), and the name of the person capturing or witnessing. Here, “witness” means witness without capture. Data of *H. bolina* ID numbers 1 to 12 (all females) were not included in the data for analyses of the sex ratio because data of adult males that were observed in the fields when the females were caught were not recorded. Therefore, including these female data in the analysis of the sex ratios could have led to data bias. Most of the analyzed data were capture data. The number of individuals of each sex was totaled in each area, or in each race, in periods of consecutive years (generally 4 consecutive years) from 1917 through 2006, and the sex ratios were calculated for these periods. An area “Kagoshima Pref. plus Okinawa Pref.” is the main area in which *H. bolina* is found in Japan (Table 3). As for the data in 2007, only the data from Okinawa Pref. (Table 3) were used in the analyses and the last was from 13 October 2007. This was because most of other potential data by others should not have been officially reported due to time lag between the observations and the report of the data.

**Table 2.**
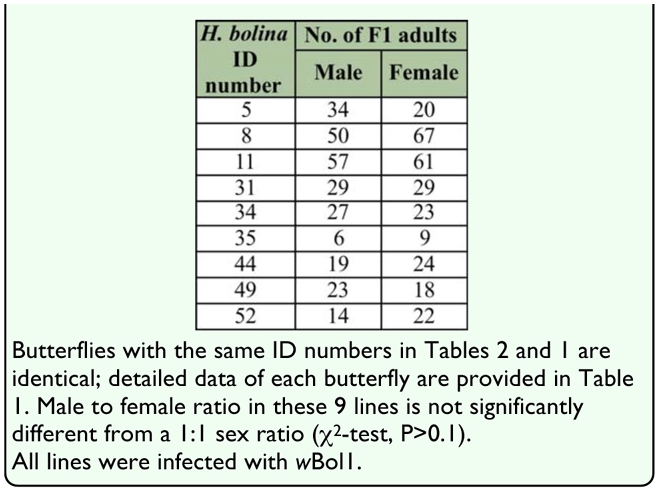
Number of males and females of F1 Progenies produced by wild adult female *Hypolimnas bolina* captured during 2004–2009

## Results

### Prevalence of recent male-killing suppression

The sex discrimination of reared F1 individuals for phenotype determination of lines is shown in Table 2. These 9 lines turned out to show an unbiased sex ratio by χ^2^ -test. In male-killing susceptible lines that were infected with *w*Boll the male:female ratios were extremely low, ranging from zero to 1:6.6, and most were zero (Clarke et al. 1975; Dyson et al. 2002; Mitsuhashi et al. 2004).

In the diagnostic PCR assays for *Wolbachia* detection, a PCR product of the expected size (ca. 920 bp) that indicates *Wolbachia* 16S rDNA was obtained from all the DNA samples. Positive and negative control PCRs functioned as expected in all of the diagnostic PCR assays. All the individuals examined were thus *Wolbachia*-positive.

In each of the adults (Table 1) examined by PCR-clone sequencing and/or direct sequencing, the sequences of *gatB, coxA, hcpA, ftsZ,* and *fbpA,* which are used for MLST for *Wolbachia,* were identical to those in the MLST of *w*Boll reported previously by Charlat et al. (2007b), with a single exception that in *H. bolina* ID number 2 another sequence was detected in some genes in addition to the *w*Boll sequence as described below. In direct sequencing, “double or multiple peaks” in the chromatograms of the sequences were not observed, with the above mentioned single exception. In addition, the sequences of *wsp* and *ftsZ* (Werren et al. 1995) that we analyzed were also identical to those of *w*Boll previously reported (Dyson et al. 2002; Mitsuhashi et al. 2004; Charlat et al. 2005, 2007b). The *w*Boll sequences determined in the present study have been deposited in the DDBJ, EMBL, and GenBank nucleotide sequence databases under accession numbers AB474245–AB474249. The sequence data have also been deposited in the Wolbachia MLST database (http://pubmlst.org/wolbachia/) under ID 270. These results thus indicate that almost all the insects examined were infected with only the one strain, *w*Boll.

**Table 3.**
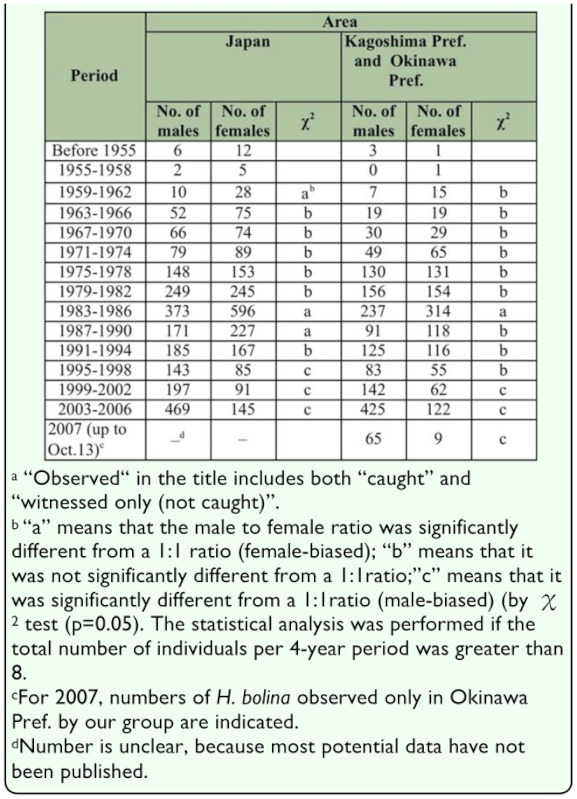
Number of each sex of wild adult *Hypolimnas bolina* observed in the indicated periods in Japan^a^ 3A. Number of H. *bolina* by area

Together, these results strongly suggest that male-killing suppression has become common in the recent populations of *H. bolina* in Japan and the areas neighboring Japan.

### A third *Wolbachia* strain in *H. bolina*

**Figure 2.**
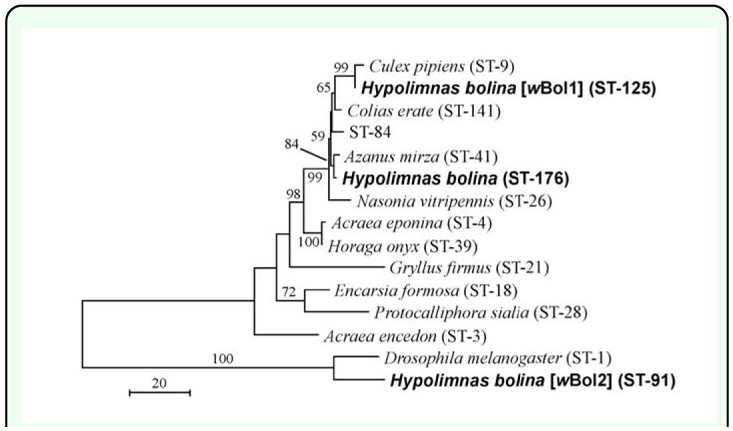
Phylogenetic tree of *Wolbachia* strains originated from various insect species. The tree was generated using the neighbor-joining method based on the numbers of nucleotide differences among taxa. *Wolbachi* strains are represented by the names of their hosts. Supergroup A strains of *Drosophila melanogaster* and *Hypolimnas bolina* are shown as outgroups. Concatenated five gene sequences for MLST were used for the analysis. Bootstrap confidence levels higher than 50% calculated based on 1,000 replications are shown near the branches. STs are sequence types in the MLST system. ST-176 was newly found in the present study. High quality figures are available online.

Clone sequencing in the sample from *H. bolina* ID number 2 revealed two types of sequences for *gatB, hcpA, ftsZ, and fbpA* and one type for *coxA.* The *coxA* sequence and one of the sequences of each of the other four genes were identical to the major type sequence (i.e. the *w*Boll sequence) obtained in the present study. However, the other sequence in each of the 4 genes was different from that of either *w*Boll or the second *Wolbachia* strain that has been identified in *H. bolina, w*Bol2 (Charlat et al. 2006). These heterogeneous sequences were also recognized by the presence of double-peaks in the direct sequencing chromatograms. Thus, these new sequences strongly suggest the presence of the third type of *Wolbachia* strain in *H. bolina.* The frequencies of the new sequences were not low: 4 of 6 *E. coli* clones harboring *gatB;* 4 of 6 *E. coli* clones harboring *hcpA;* 2 of 6 *E. coli* clones harboring *ftsZ*; and 4 of 6 *E. coli* clones harboring *fbpA.* Also, in each of the double-peaks of the direct sequencing chromatograms, the height of one peak was in most cases similar to the height of the other peak. Judging from these 2 facts, the amount of each type of DNA molecule in each PCR product appeared to be similar, suggesting that both types of *Wolbachia* coexisted in similar numbers in the excised ovaries. Moreover, no sequences found in the GenBank and MLST databases were identical to the *hcpA* sequence of the third type *Wolbachia,* and the MLST typing (sequence type (ST)-176, ID 271) of the third type *Wolbachia* was also new to the MLST database. The *ftsZ* sequence for MLST of the third type *Wolbachia* showed 99.8% and 90.1% identity with those of *w*Boll and *w*Bol2, respectively. A phylogenetic tree constructed by the neighbor-joining method strongly suggests that the *Wolbachia* with the new sequences is a strain different from *w*Boll, although it is closely related genetically to *w*Boll (Figure 2). The new *Wolbachia* sequences have also been deposited in the nucleotide sequence databases under accession numbers AB513352–AB513355 and AB516429.

### Analyses of capture and witness data over the past 50 years

**Figure 3.**
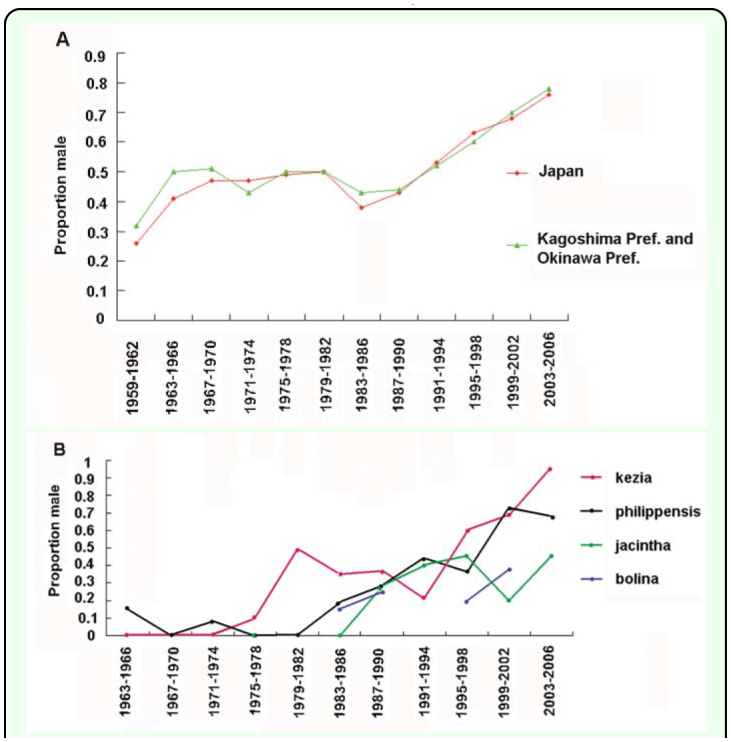
Proportion of males in the total number of wild adult *Hypolimnas bolina* observed in Japan in each 4-year period over the past approximately 50 years from 1959 through 2006. (A) The status of the proportion of males in the major distribution area of Japan, i.e. Okinawa and Kagoshima Prefectures, and the whole of Japan was analyzed. (B) Status of the proportion of males in each race was analyzed. The proportions of males in (A) and (B) were calculated based on the number of *H*. *bolina* adults shown in Tables 3A and 3B, respectively. The proportion of males was not calculated if total number of individuals per 4-year period was less than 8. High quality figures are available online.

The results of the analyses are shown in Table 3 and Figure 3. In summary, the number of adult males as a proportion of the total number of adults (hereafter, “proportion of males”) in the species as a whole in Japan has risen with time with an apparent stagnation in the middle of the period (Table 3A, Figure 3A). An increasing proportion of males was also observed in the races kezia, philippensis, and jacintha; although the increase in jacintha was not as distinct as those in the other two races (Table 3B, Figure 3B). Thus the increase in the proportion of males in the species should be mainly due to the increase in males in the races kezia and philippensis. In 2007, which was the last year analyzed, the proportion of males in the whole of the species reached 0.88. Data for the months of 2007 are: January 1♀; February 0; March 3♂; April 1♂; May 2♂; June 2♂ and 2♀; July 13♂; August 15♂ and 3♀; September 10♂ and 1♀; October (up to 13 October) 19♂ and 2♀.

The large quantity of data gathered over this long period should compensate for defects arising from the lack of uniformity in the methods of data collection in the field over the past 50 years. The proportion of males calculated by race is likely to be somewhat lower than what could be observed in fields (Table 3; Figure 3). This is to be expected because the differences in external appearance of the wings of males of different races are not as apparent as those of female wings, and thus it is more difficult to identify the races of males than it is to identify the races of females, especially in the wild, and for this reason the race would not be identified as frequently for males as it would be for females.

## Discussion

The historical results in the present study strongly suggest that male-killing suppression lines have generally increased in abundance in Japan over the past 50 years. Among the 4 races in Japan - races kezia, philippensis, and jacintha have shown an increase in the proportion of males during the past 50 years, although jacintha has only shown a moderate increase. The trend in race bolina was not clear because of the small sample size. However, the fact that race bolina in the South Pacific still contains many male-killing susceptible lines (Charlat et al. 2005, 2006, 2007b) suggests that the trend for race bolina in Japan was similar to that in the South Pacific. Thus, these results suggest that this increase in the ratio in the species as a whole in Japan appears to be mainly due to an increase in the suppression of male-killing in races kezia and philippensis.

The experimental analyses of the *H. bolina* population from 2003–2009 in Japan strongly suggest that male-killing suppression has recently become common in the population. This is supported by the following: (1) all the adult males we examined harbored *w*Boll; because males infected with *w*Boll in male-killing susceptible lines die in the early stages of their development, any adult male possessing *w*Boll will turn out to be a member of an male-killing suppression line (Charlat et al. 2005; Hornett et al. 2006); (2) each of the lines with *w*Boll that were examined were male-killing suppression lines, as determined by sexing the F1 adults; in addition to the 9 lines shown in Table 2, we observed the sex ratio of the F1 adult offspring of another 5 adult females (*H. bolina* ID numbers 1–4 and 7 in Table 1) collected in the wild in Okinawa in 2003 and 2004, which turned out to be *w*Boll-positive in the present study. As was the case with the former 9 females, these 5 females had also oviposited without mating after capture. Although the precise number of respective sexs of F1 adults was not formally recorded, there were plentiful F1 adult males compared to the number of F1 adult females, indicating that these 5 lines were also male-killing suppression lines. Thus, all 14 lines were male-killing suppression lines. The historical results together with the recent status in the *H. bolina* strongly suggest that male-killing suppression lines have generally continued to increase over the past 50 years in Japan. To confirm this conclusion, it would be desirable to conduct crossing tests between a Japanese *w*Boll-infected line with a normal sex ratio and a male-killing susceptible line from somewhere and investigate the prevalence of *w*Boll in old Japanese host population.

**Table 3 3B.**
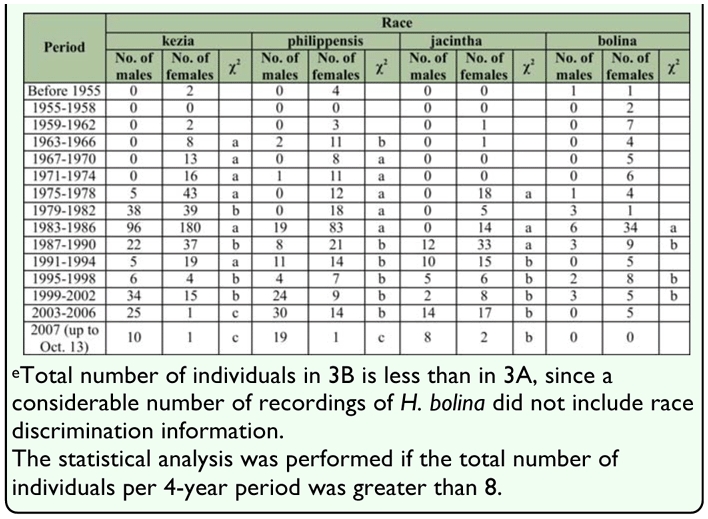
Number of *H. bolina* by race^e^

In general, even if the sex ratio in a butterfly population is 1:1, adult male butterflies tend to be found in the wild more easily than females because males are generally more active than females (Matsumoto 1984; Kobayashi and Inaizumi 2003); this tendency is also the case in *H. bolina* (Kitamura 1999). Therefore, in a population with a 1:1 sex ratio, more adult *H. bolina* males than females would be expected to be caught or witnessed in the wild and the proportion of males should be far greater than 0.5. Recent values far greater than 0.5, therefore, do not indicate male-biased sex ratios evolving in the population, and this is supported by the significant 1:1 sex ratio of the reared insect lines shown in Table 2. Therefore the proportions of males of approximately 0.5 and below 0.5 prior to 1990 found in the present study strongly suggests that the Japanese population prior to 1990 contained a considerable number of male-killing susceptible lines. For example, the existence of female-biased lines of *H. bolina* in Japan, which was revealed by the sex ratio of their adult offspring (F1), was reported numerous times before 1975 (Kawazoe and Wakabayashi 1976).

The prevalence of male-killing in *H. bolina* in Japan at a particular time should strongly reflect the status of male-killing in the butterfly populations in areas neighboring Japan, such as the Philippines, Formosa, the southern part of China, and the Indochinese Peninsula during the same period. Other reports have implied the prevalence of male-killing in the Philippines. The proportion of males was only approximately 7% of the 1623 *H. bolina* adult individuals collected before 1893 in the Philippines (excluding the islands of Palawan) (Semper 1892), and this suggests that the male-killing trait was formerly common there. In contrast, two reports have suggested the recent predominance of male-killing suppression lines; the sex ratio (male:female) of adult *H. bolina* caught from June 1997 through December 1998 in an area of Luzon Island in the Philippines was approximately 3:1 (Kitamura 1999), and introgression of genes from 2 lines collected from the Philippines onto male-killing susceptible lines in Polynesia confirmed that the 2 lines from the Philippines were male-killing suppression lines (Hornett et al. 2006). These 3 reports thus suggest a parallel change in the status of male-killing in the species in the Philippines to that in the status of it in Japan. It was reported that the male-killing suppression was common in *H. bolina* population in the Philippines around 1900 based on the high rate of detection of *w*Boll sequences in museum male-specimens (Hornett et al. 2009). The reasons for the discrepancy between the data obtained by Hornett et al. (2009) and those in the present study are unclear, but it may be that the prevalence of male-killing might be different among islands at that time and that migrations to Japan may have been primarily from populations on only some island(s) where male-killing susceptible lines were distributed, since the Philippines is a large area and composed of numerous islands.

The suppressor of male-killing seems to have started spreading from some distributional area of races kezia and philippensis (i.e. southern China for kezia or the Philippines for philippensis). Four percent of 212 adults collected during June 1997 to December 1998 in an area of Luzon Island were of the race, kezia, based on the external features of the wings (Kitamura 1999); strongly suggesting that the kezia race has mated with the philippensis race and there has been a transfer of the male-killing suppression trait between the two races. We therefore, hypothesize that the origin of *H. bolina* harboring the male-killing suppressor was populations of the philippensis race in the Philippines or of the kezia race in Formosa or the southern part of continental China.

The third type of *Wolbachia* sequences of *H. bolina* in a single individual (*H. bolina* ID number 2) has been detected in the present study. There is little possibility that parasitoid or mite DNA with its symbiotic *Wolbachia* DNA contaminated the DNA sample, since the ovaries were carefully excised from the abdomen under a binocular microscope on a clean-bench as described in the Materials and Methods section. The phylogenetic tree strongly suggests that the *Wolbachia* with the new sequences is a new strain that is closely related genetically to *w*Boll. Thus, we conclude that a third *Wolbachia* strain in *H. bolina* co-existed with *w*Boll. The F1 of *H. bolina* ID number 2 showed a normal sex ratio as mentioned above, although the precise number of each sex was not recorded, indicating the adult female was a member of a male-killing suppression line. It would be interesting to conduct further research to elucidate whether the third *Wolbachia* strain induces a phenotype of reproductive alteration in a host or not.

In the present study, the interaction between a reproductive parasite and its host species was analyzed in a natural population over a continuous 50-year period, which is a much longer period than has previously been examined. The results of this analysis strongly suggest a long-term trend of increasing host resistance to the action of their symbionts in natural populations. These results therefore represent an instance of evolutionary flexibility in host-symbiont interactions. The present study was made possible by the existence of numerous data collected over a long and continuous period; data covering such a long and continuous period of years are likely to be rare for most other species.

## Abbreviations

MLST,: multilocus sequence typing;
ST,: sequence type
